# Supplementary figures and images for: Role of the Ca2+ channel α2δ-1 auxiliary subunit in proliferation and migration of human glioblastoma cells
Source: PLoS One. 2022 Dec 15;17(12):e0279186. doi: 10.1371/journal.pone.0279186 (PMC9754164; doi:10.1371/journal.pone.0279186)

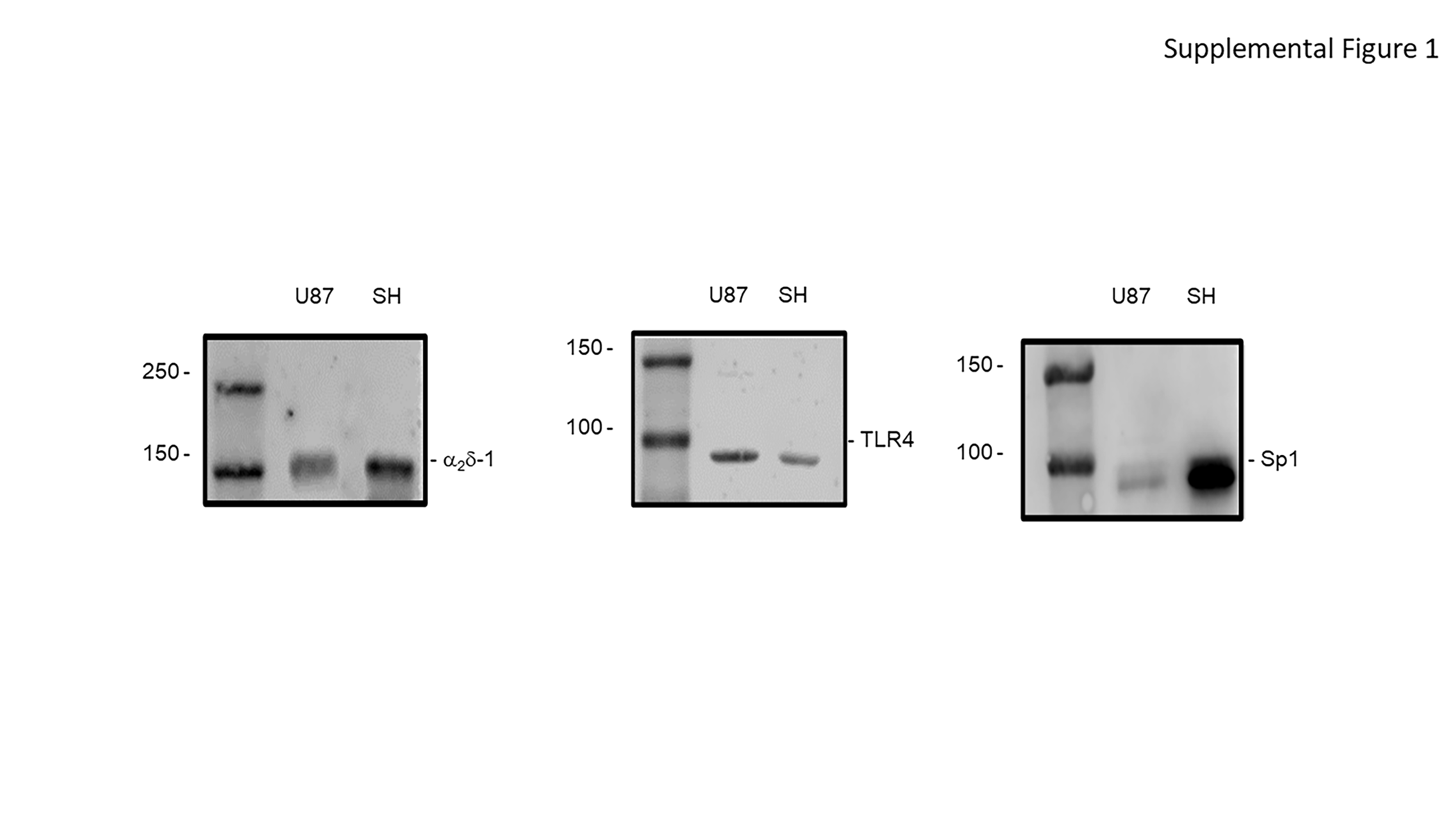

Supplement: S1 Fig — The image shows representative blots of three experiments performed separately. The signal obtained with the β-actin antibody served as the loading control. (TIF) [file pone.0279186.s001.tif]

Figure 1

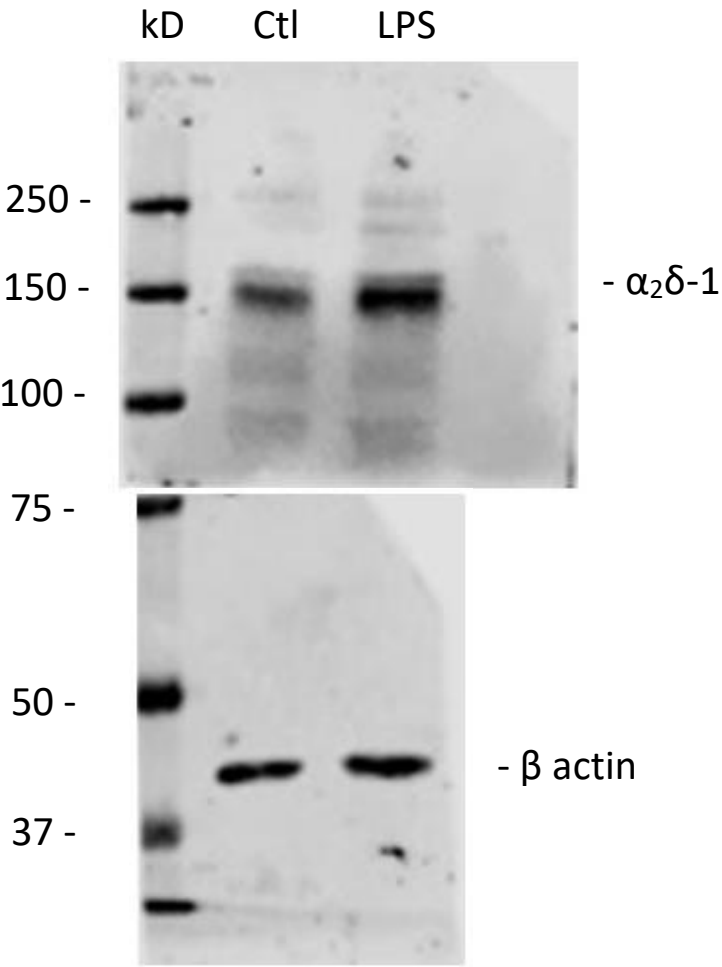

Figure 3

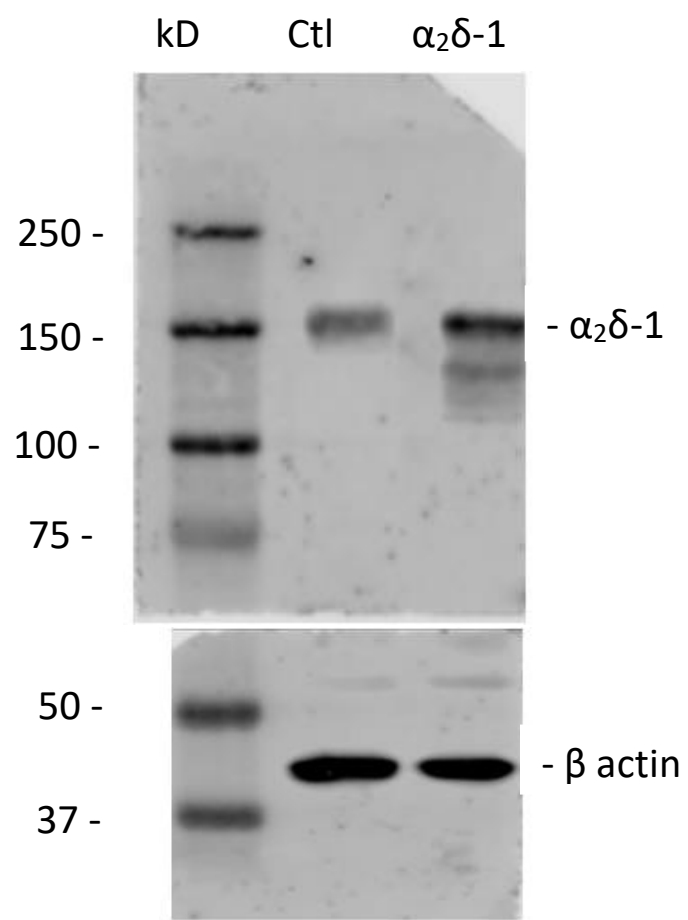

Figure 8

**A**

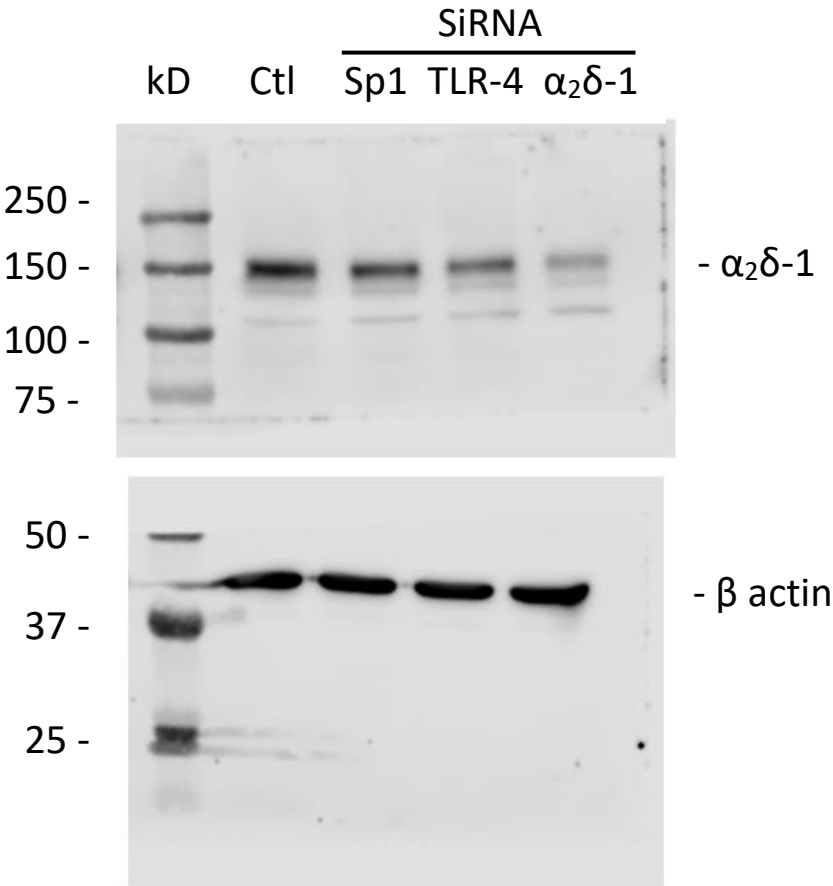

**B**

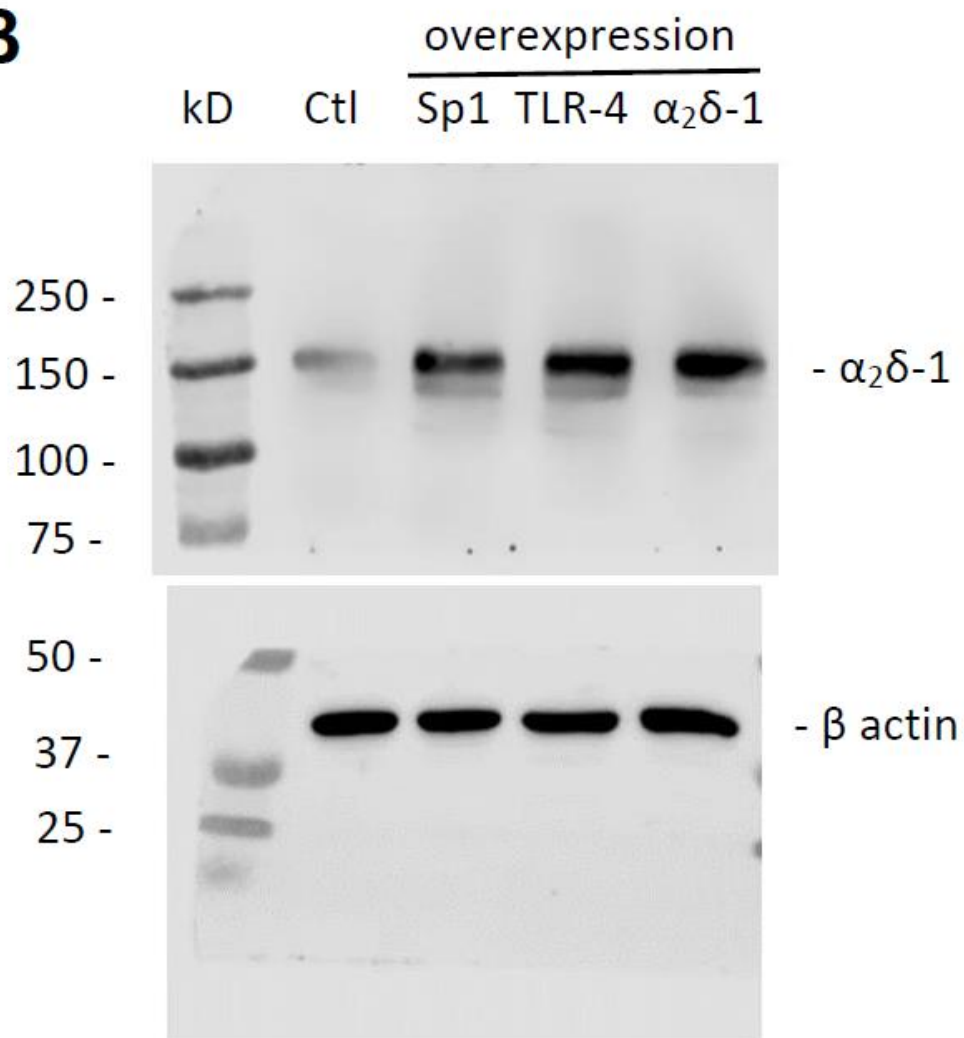

Figure 9A

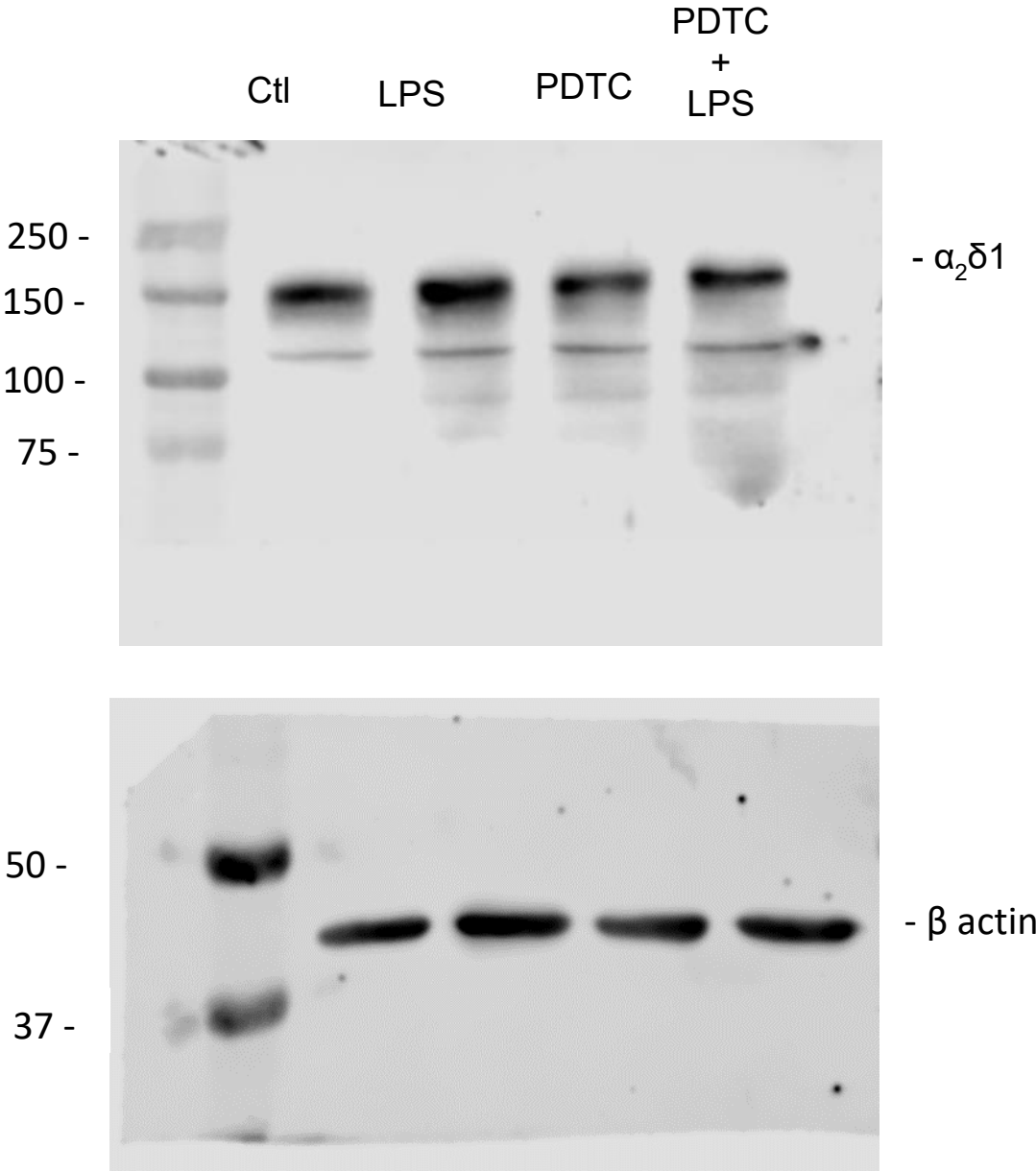

Figure 9B

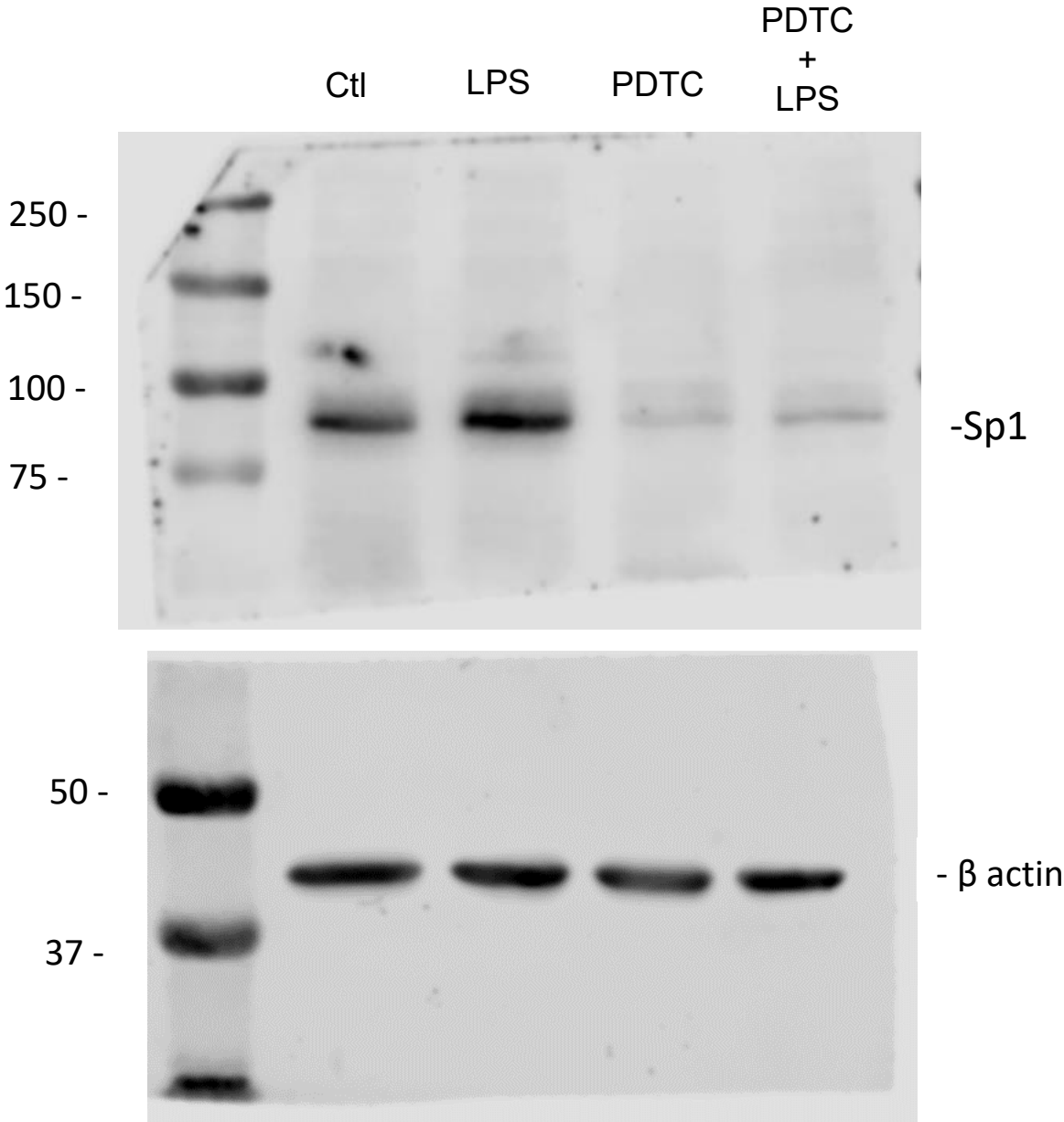

Supplemental Figure 1

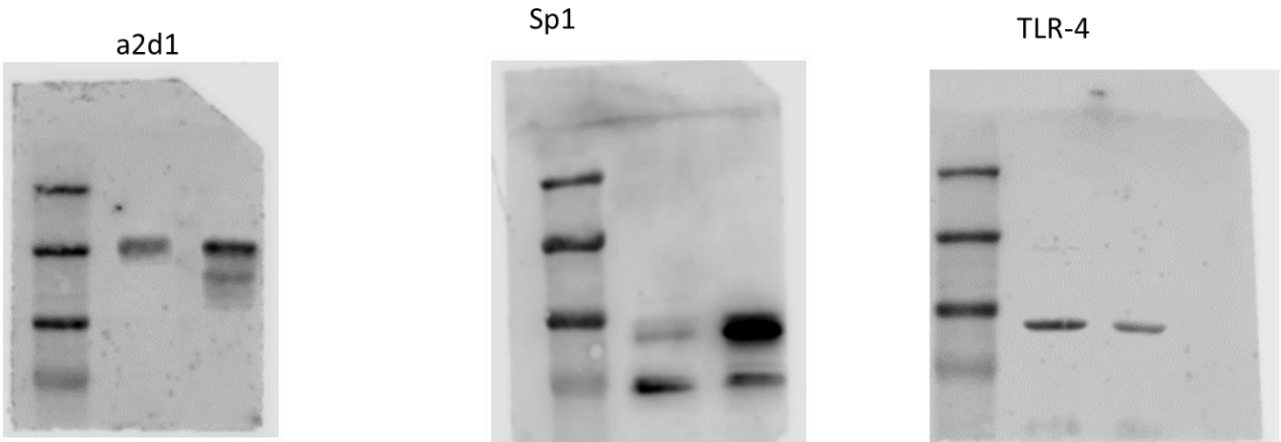

Supplement: S1 Raw images — (PDF) [file pone.0279186.s002.pdf]
