# Supplementary material for: Role of the Ca2+ channel α2δ-1 auxiliary subunit in proliferation and migration of human glioblastoma cells
Source: PLoS One. 2022 Dec 15;17(12):e0279186. doi: 10.1371/journal.pone.0279186 (PMC9754164; doi:10.1371/journal.pone.0279186)
Supplement: Attachment — Submitted filename: Response to Reviewer.docx [file pone.0279186.s003.docx]

**Response to Reviwer’s comments:**

Reviewer #1:

Comment 1.

The data suggest a direct action of α2δ1 protein expression as response to LPS stimulation. To further narrow the specificity of LPS induced proliferation a positive control via the application of Thrombospondin-1 would strengthen the correlation between the stimulus and the impact of α2δ1-proteins as receptor molecule of Throbospondin-1. Since Thrombospondins are also for neurons relevant ECM molecules that influence synaptogenesis, such experiment could hint to specific possible interactions between glioma cells and their cellular environment.

Response to Comment 1.

As the reviewer points out, the use of Thrombospondin-1, a ligand of the a2d-1 subunit, would help to strengthen the proposal that this protein could significantly contribute to determining the proliferation and migration processes in the cell line derived from glioblastoma. In fact, following the reviewer's suggestion, we have performed additional experiments to evaluate the effects of Thrombospondin-1 on the proliferation and migration of U87 cells. Our results suggest that incubation with Thrombospondin-1 for 24 h significantly increases both cellular processes. This analysis has been included as a new figure (see Fig 5) in the revised version of the manuscript. Accordingly, a detailed description of this new series of experiments has been included in both the Results section (p. 12; l. 246-255) sections. We thank the reviewer for the suggestion that has undoubtedly strengthened our interpretation that a2d-1 participates in cell proliferation and migration processes.

Comment 2.

Within the western blot analysis of expression, the data have been normalized to the control, but the controls missing an indication of the standard error. To better interpret the significance of the effects such information would be very helpful to see how reliable/stable are the western blot experiments. Please include such error information into the graphs.

Response to Comment 2.

Corrected. In order to provide more information on the interpretation of the results, following the reviewer's suggestion in the manuscript version, the error bars for the control condition have been included in all bar graphs included in the paper (see Figs 1A and 1C; Figs 2A, 2B, 2D; Fig 3; Fig 4B; Figs 5A and 5C; Figs 6A and 6C; Figs 7A and 7C; Figs 8A and 8B; Figs 9A and 9B). Once again, we thank the reviewer for the suggestion.
